# Spatial patterns and influencing factors of traditional villages in the Pearl River–Xijiang River Economic Belt (PRXREB)

**DOI:** 10.1371/journal.pone.0321646

**Published:** 2025-04-18

**Authors:** Meng Dai, Xiuli Huang, Yuanquan Xu, Zhibo Han

**Affiliations:** 1 School of Physical Education and Health, Guangxi Normal University, Guilin, Guangxi, China; 2 Communist Youth League Committee, Guangxi Normal University, Guilin, Guangxi, China; 3 School of Environment and Resources, Guangxi Normal University, Guilin, Guangxi, China; 4 Guangxi Key Laboratory of Environmental Processes and Remediation in Ecologically Fragile Regions, Guangxi Normal University, Guilin, Guangxi, China; Macau University of Science and Technology, MACAO

## Abstract

The spatial patterns and influencing factors of traditional villages are critical for their preservation and heritage. This study employs spatial analysis and geostatistical methods to explore the spatial distribution of national traditional villages within the PRXREB as of 2023. The results indicate an uneven and clustered distribution, identifying four distinct clusters within the region. The analysis shows that most traditional villages are located near major rivers and roads, as well as in areas with slopes less than 10°. Importantly, the distribution of traditional villages in the PRXREB is shaped by the interplay of multiple factors rather than isolated individual factors, the complex interplay between natural factors and socio-economic conditions likely shapes the sustainable development of traditional villages by affecting long-term economic development path. Particularly, some traditional villages in Guangxi with challenging natural environments face risks due to population loss and inadequate transportation infrastructure. We find that the spatial distribution of traditional villages is significantly positively correlated with the spatial distribution of regional population and various economic indicators. The per capita disposable income of urban residents (PIUR) is the most influential factor, and economic development helps promote the protection and inheritance of traditional villages, but there are also regional differences. Based on these insights, we propose targeted recommendations to support the sustainable development and conservation of traditional villages in the PRXREB.

## 1. Introduction

Traditional villages hold significant historical, landscape, and cultural value, serving as vital representations of rural civilization and settlement forms. However, the rate of urbanization worldwide has reached 56%, with a consistent annual increase in most countries [1]. Within the context of the urban-rural dual structure system, the aggressive expansion of cities has led to rural depopulation and marginalization. The decline and loss of heritage in traditional villages are becoming increasingly severe. Understanding the spatial distribution of traditional villages and their natural, social, and economic environments is crucial for their revitalization. Therefore, studying the spatial layout and developmental factors influencing traditional villages has become essential for their sustainable development and protection.

Current studies on the traditional villages can be divided into three categories. The first category focus on spatial analysis of traditional village distributions. Utilizing GIS and remote sensing technologies, these studies map and analyze the distribution and clustering of villages [2]. Such methods help delineate patterns and clusters related to natural factors. Some researchers have conducted detailed studies on the spatial distribution of traditional villages at regional [2,3], provincial [4–6], and county levels [7–9], with some studies focusing on typical villages in micro-units [10–13]. For example, Wei et al found that The traditional villages in the Yellow River Basin exhibit an east-west density gradient, with a higher concentration in the east and sparser distribution in the west [2]. The spatial dynamics, spatial pattern, and morphological characteristics of traditional villages in different province exhibit clear geographic imbalances, with the impacts of various driving factors differing significantly [4–6]. Similarly, the spatial distribution of traditional villages across counties is uneven, with small and medium-sized villages predominating [7–9]. At the micro-scale, scholars primarily focus on the spatial characteristics and development of traditional villages, heritage conservation, landscape evaluation, rural tourism, and ecological planning [10–12]. They suggest that rural planning and design should be based on current conditions, with an emphasis on strengthening public infrastructure planning, adapting to local conditions, and realizing bottom-up development [13]. Although existing research has analyzed the distribution characteristics and driving mechanisms of traditional villages at multiple levels, most studies focus on a single river basin or province as the study area. Few basin-specific studies primarily concentrate on the Yellow River Basin and the southwestern mountainous areas of China, and there is a lack of research on the cultural-ecological interaction mechanisms in the China-ASEAN corridor.

The second category examines the socio-economic impacts on village spatial distributions. This strand of research explores the impact of economic policies and migration on the sustainability of rural villages. The socio-economic factors include architectural space [14,15], settlement culture [16], landscape patterns [17], community livelihood [18], medical environment [19], urban–rural relationship and social mobility [5,20], etc. Econometric models and demographic analyses assess the effects of urban expansion on rural depopulation and cultural erosion. However, many studies overlook the bidirectional nature of socio-economic dynamics and their spatial manifestations, limiting their applicability to broader policy contexts.

The third category assesses the effectiveness of heritage preservation strategies within traditional villages [6,21]. Revitalizing rural areas is now a key global strategy in addressing these challenges. For example, Germany has adopted a multifaceted approach, including land consolidation, area planning, rural infrastructure enhancement, and educational initiatives, to boost rural resilience and revitalization [22]. In contrast, Japan, Thailand, and Vietnam have implemented the “One Village, One Product” initiative to stimulate rural industrial growth and revitalization [23–25]. The United States is focusing on fostering community dynamism as a means of rural revitalization [26]. Sweden, meanwhile, is advancing rural revitalization by introducing external social capital to enhance rural productive capacities [27]. In China, rural revitalization strategies involve blending rural and urban development and promoting new models of urbanization, particularly through the branding of traditional villages. These qualitative methods, including ethnographic studies and community-based participatory research, provide insights into successful preservation initiatives. However, these approaches tend to be localized and may not scale well across different regions.

Despite the Chinese government recognition of 8,171 national traditional villages release in 2023, their disappearance rate surpasses their acknowledgment, highlighting the urgency of their conservation [28]. As the first cross-provincial basin economic belt, the Pearl River–Xijiang River Economic Belt (PRXREB) is strategically significant for the transformation and development of the Pearl River Delta, linking the developed eastern regions with the less developed western regions, particularly in the Guangxi Zhuang Autonomous Region and Guangdong Province. The regional differences within this economic belt have made it a focal point for multidisciplinary scholarly research [29–31]. Investigating the spatial distribution and influencing factors of traditional villages in the PRXREB, identifying the bottlenecks in rural development, and providing scientific bases for conservation strategies are vital for preserving the cultural, landscape, and historical heritage of this region [32,33].

In our study, 120 national traditional villages within the 11 cities of the PRXREB are selected as the research objects. We aim to merge spatial and socio-economic perspectives to offer a comprehensive understanding of the dynamics affecting traditional villages in the PRXREB. Our goal is to develop targeted recommendations that facilitate both cultural heritage preservation and sustainable development [34]. We seek to provide a new perspective on regional development in the PRXREB, offer theoretical support and practical guidance for the understanding and protection of traditional villages, and provide scientific evidence for local governments and policymakers, helping them balance economic development with traditional village conservation and promoting regional sustainable development. The structure of the paper is as follows: Section 2 describes the data and methodology, Section 3 presents the spatial patterns of traditional villages in the PRXREB, Section 4 analyzes the influencing factors of traditional villages in the PRXREB, Section 5 discusses the findings and policy recommendations, and Section 6 concludes with results and future research directions.

## 2. Materials and methods

### 2.1. Study area

Located in the Pearl River Basin in the south of China, the PRXREB borders Yunnan Province and Guizhou Province in the north, crosses Guangdong and the Guangxi Zhuang Autonomous Region, and connects Hong Kong and Macao in the south. The PRXREB contains four cities in Guangdong Province, namely, Guangzhou, Foshan, Zhaoqing, and Yunfu, and seven cities in the Guangxi Zhuang Autonomous Region, namely, Nanning, Liuzhou, Wuzhou, Guigang, Baise, Laibin, and Chongzuo, and it covers 0.165 million km^2^, accounting for 6.6% of the national territory of southwest China (2.5 million km^2^). Moreover, the PRXREB is located in the western part of the hills of the Guangxi Zhuang Autonomous Region and Guangdong Province, with its high terrain in the northwest and low terrain in the southeast; the terrain is inclined from the northwest to the southeast, and the landform is generally composed of mountains, hills, plains, and rivers. The mountains and hills account for 53.88% of the total area of the PRXREB. There are many mountains in the western region, a dense network of rivers in the east-central part, and a lower altitude and flat terrain in the Pearl River Delta.

Now, the PRXREB is inhabited by several ethnic groups in compact communities, including the Hans, the Miaos, the Yaos, the Dongs, and the Zhuangs, and most of the ethnic minorities live in the Guangxi Region. Among the 120 national traditional villages in this economic belt, there are 50 villages in Guangdong Province and 70 villages in the Guangxi Region. Although the spatial distributions of these traditional villages have certain similarities, in Guangxi and Guangdong, there is a huge gap between the human environment and economic development, as well as in the characteristics of the living preservation and development of traditional villages. As the direct hinterland of the Guangdong–Hong Kong–Macao Greater Bay Area, the PRXREB has an important strategic position in the national coordinated regional development and the opening up and cooperation with ASEAN, as it is an important link for the southwest region and even the Southeast Asia and South Asia regions driven by the Guangdong–Hong Kong–Macao Greater Bay Area, as well as a sea channel for the southwest region of China.

### 2.2. Data sources

Data on the traditional villages located in the PRXREB were obtained from the website of Chinese Traditional Villages (source: http://www.chuantongcunluo.com/index.php/Home/gjml/gjml/wid/2506.html, accessed on 8 Sep 2023), and the coordinates of those villages were collected from the National Platform for Common GeoSpatial Information Services (https://www.tianditu.gov.cn/, accessed on 8 Nov 2024). A digital elevation model (DEM) was derived from Aster GDEMV3 data (spatial resolution: 30 m) provided by the NASA Earthdata (source: https://search.earthdata.nasa.gov/search, accessed on 10 Nov 2024). The spatial distribution of the population in the PRXREB was obtained from WorldPop (spatial resolution: 100 m, source: https://www.worldpop.org/, accessed on 15 Sep 2023). River and road network data were downloaded from the Open Street Map (source: https://www.openstreetmap.org/, accessed on 20 Sep 2023). In addition, economy data were collected from the China statistical yearbooks and government reports of relevant cities in the PRXREB. Table 1 describes the study data in detail.

**Table 1:** Description of the data.

Data	Source	Year	Specific uses
Traditional villages	Chinese Traditional Villages(http://www.chuantongcunluo.com/index.php/Home/gjml/gjml/wid/2506.html)	2023	The name of the village and the province and city where it is located
Coordinates of traditional villages	the National Platform for Common GeoSpatial Information Services (https://www.tianditu.gov.cn)	2024	Spatial location of the village (S1 Table)
DEM	ASTER Global Digital Elevation Model V003, NASA Earthdata (https://search.earthdata.nasa.gov/search)	2013	The elevation, slope and RDLS of the village
Population	WorldPop (https://www.worldpop.org)	2020	Population of the village
Road and River	Open Street Map (https://www.openstreetmap.org)	2023	The distance from villages to rivers and roads
Economy	China statistical yearbook (S2 Table)	2023	Economy of the city where the village is located

### 2.3. Research methods

**Fig 1:**
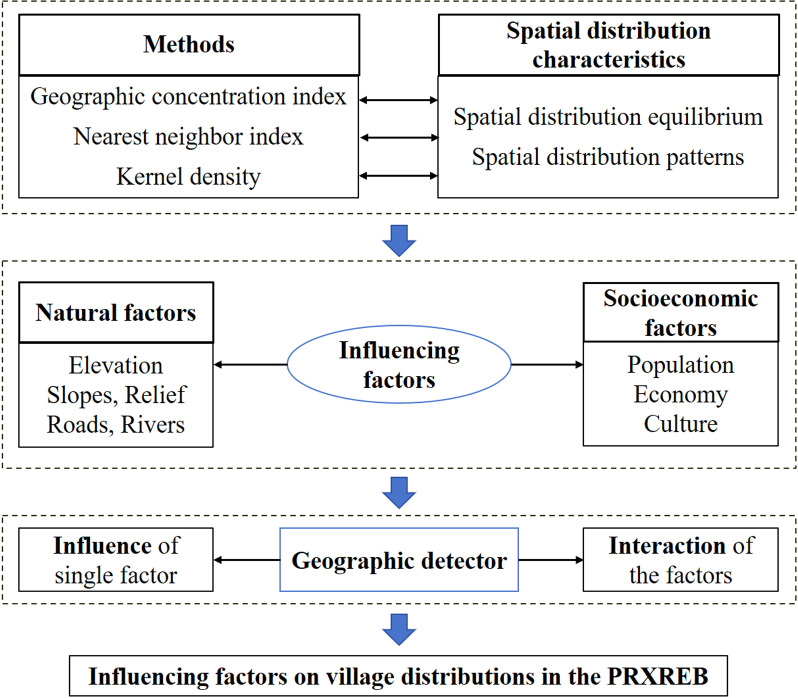
The framework of this study.

In our study, geographical indicators, such as the nearest neighbor index, geographic concentration index, kernel density estimation, and the geographic detector, were mainly used to characterize the spatial variations in the traditional villages in the PRXREB, and the influences of the terrain, roads and transportation, rivers, and economic development on the spatial distributions of the study objects were explored. The framework of this study is shown in Fig 1.

#### 2.3.1. Geographic concentration index.

The geographical concentration index can be used to measure the concentration of traditional villages in different cities in the PRXREB. The calculation formula is


$$\begin{array}{c} G=100\times \sqrt{\sum_{i=1}^{c}{\left(\frac{x_{i}}{n}\right)}^{2}} \end{array}$$ (1)

where G is the geographical concentration index, which ranges from 0 to 100. c is the number of cities. $x_{i}$ is the number of traditional villages in city i. n is the total number of traditional villages in the PRXREB. If the geographical concentration index of a city is greater than that when traditional villages are evenly distributed in the cities of the economic belt, then it means that the traditional villages in each city are concentrated; otherwise, they are dispersed.

#### 2.3.2. Nearest neighbor index.

The nearest neighbor index [35] is defined as the ratio of the observed mean distance to the expected mean distance of the traditional villages, which can measure the proximity of the traditional villages in geographic space. Its formula is as follows:


$$\begin{array}{c} R=\frac{{\overset{―}{D}}_{O}}{{\overset{―}{D}}_{E}} \end{array}$$ (2)

where R is the nearest neighbor index. If R>1, the distribution of traditional villages is uniform. If R<1, the distribution of traditional villages is clustered. If R=1, the distribution of traditional villages is random. ${\overset{―}{D}}_{O}$ is the observed mean distance between each traditional village and its nearest neighbor. ${\overset{―}{D}}_{E}$ is the expected mean distance between each traditional village and its nearest neighbor. When the traditional villages in the PRXREB fit the Poisson distribution, ${\overset{―}{D}}_{E}$ is calculated as follows:


$$\begin{array}{c} {\overset{―}{D}}_{E}=0.5\sqrt{\frac{A}{n}} \end{array}$$ (3)

where n is the total number of traditional villages, and A is the area under study.

#### 2.3.3. Kernel density estimation.

The kernel density estimation [36] uses a moving window to estimate the density of the traditional villages, and it can identify the clusters of the traditional villages in the PRXREB. The calculation formula is


$$\begin{array}{c} f(x)=\frac{1}{nh}\sum_{i=1}^{n}K\left(\frac{x-x_{i}}{h}\right) \end{array}$$ (4)

where f(x) is the estimated density of the traditional village at x. The larger the value of f(x), the denser the traditional village. $x_{i}$ represents the coordinates of the traditional village. n is the number of traditional villages. h is the search bandwidth. $K\left(\frac{x-x_{i}}{h}\right)$ is the kernel function.

#### 2.3.4. Geographic detector.

The geographic detector [37] is a geostatistical method for exploring the relationship between the factors and spatial patterns of t she traditional villages, and it includes 4 detectors: the factor detector, risk detector, ecological detector, and interaction detector. We applied the factor detector and interaction detector to explore the importance of each factor and how different factors interact with each other.

The factor detector can quantify the importance of the factors in the spatial pattern of the traditional villages. The calculation formula is


$$\begin{array}{c} q=1-\frac{\sum_{h=1}^{L}N_{h}\sigma_{h}^{2}}{N\sigma^{2}} \end{array}$$ (5)

where q is between 0 and 1, indicating the consistency of the spatial distribution between traditional villages and its influencing factors. The larger the value of q, the more important the factor in the spatial pattern of the traditional villages. q=1 means that the factor completely controls the spatial pattern of the traditional villages, and q=0 means that the factor has no relationship with the spatial pattern of the traditional villages. L is the number of the influencing factor’s strata. $N_{h}$ and $\sigma_{h}$ are the number of and the variance in the traditional villages at strata h. N and σ are the numbers of and the variance in all traditional villages in the PRXREB.

The interaction detector can explore the interaction between different influencing factors on the spatial pattern of traditional villages and quantify the importance of the factors in the spatial pattern of the traditional villages. By comparing the q-value of the interaction of two different factors with the q-value of a single factor, the interaction detector can be classified into 5 types:


$$\begin{array}{c} \left\{ \begin{array}{c} \begin{array}{cc} Nonlinear-weaken: & q\left(x_{i}\cap x_{j}\right)<Min\left(q\left(x_{i}\right),q\left(x_{j}\right)\right) \end{array}\\ \begin{array}{c} \begin{array}{cc} Uni-enhance\text{/}weaken: & Min\left(q\left(x_{i}\right),q\left(x_{j}\right)\right)<q\left(x_{i}\cap x_{j}\right)<Max\left(q\left(x_{i}\right),q\left(x_{j}\right)\right) \end{array}\\ \begin{array}{cc} Bi-enhance: & Max\left(q\left(x_{i}\right),q\left(x_{j}\right)\right)<q\left(x_{i}\cap x_{j}\right)<q\left(x_{i}\right)+q\left(x_{j}\right) \end{array}\\ \begin{array}{c} \begin{array}{cc} Independent: & q\left(x_{i}\cap x_{j}\right)=q\left(x_{i}\right)+q\left(x_{j}\right) \end{array}\\ \begin{array}{cc} Nonlinear-enhance: & q\left(x_{i}\cap x_{j}\right)>q\left(x_{i}\right)+q\left(x_{j}\right) \end{array} \end{array} \end{array} \end{array}\right. \end{array}$$ (6)

where $q\left(x_{i}\right)$ is the value of the interaction of two different factors, $x_{i}$ and $x_{j}$, and it is calculated using Equation 5. $q\left(x_{i}\right)$ and $q\left(x_{j}\right)$ are the values of factors $x_{i}$ and $x_{j}$, respectively.

## 3. Spatial distribution characteristics of traditional villages in the PRXREB

### 3.1. Spatial distribution equilibrium

**Fig 2:**
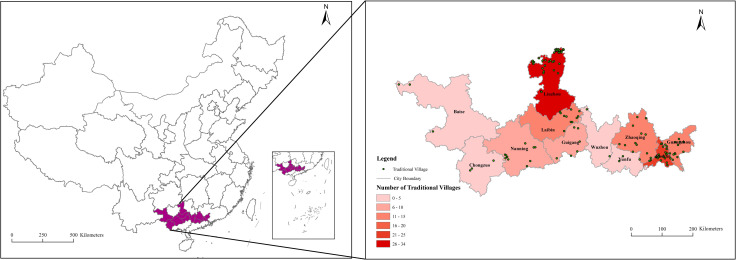
The spatial distribution of traditional villages in the PRXREB. (Source of base map: the open source map data service provided by the National Platform for Common GeoSpatial Information Services (https://www.tianditu.gov.cn/).

In our study, traditional villages in the PRXREB are presented as spatially visualized points. The statistics of the traditional villages in the PRXREB are calculated and graded in Fig 2. According to the data shown in Fig 2, the spatial distributions of the traditional villages in the PRXREB are unbalanced and vary among cities. Specifically, the spatial distribution characteristics of the traditional villages are dominated by two major clusters: one is Liuzhou, Laibin, and Nanning in Guangxi Province, accounting for 46.67% of the total number of traditional villages in the PRXREB, and the other is Foshan, Guangzhou, and Zhaoqing in Guangdong Province, accounting for 39.17% of the total number of traditional villages in the PRXREB. The distributions of the traditional villages in the other cities are relatively dispersed.

Through the geographical concentration index, the spatial distribution equilibrium of the traditional villages can be quantified. According to Equation 3, geographic concentration index G of the traditional villages in the PRXREB is 39.45; however, if 120 traditional villages are evenly distributed in each city in the PRXREB, the geographic concentration index $G^{\prime }$ is 30.15. $G>G^{\prime }$ indicates that the spatial distribution of the traditional villages in the PRXREB is unbalanced and agglomerated.

### 3.2. Spatial distribution patterns

The result of the nearest neighbor index of the traditional villages in the PRXREB is shown in Table 2. Among the traditional villages in the PRXREB, the average distance ${\overset{―}{D}}_{O}$ reaches 12.66km, while the expected theoretical distribution average distance ${\overset{―}{D}}_{E}$ is 27.14km. The nearest neighbor index (\textit{R}) is 0.47, which is less than 1 and passes the significance test, indicating that the spatial distribution of the traditional villages in the PRXREB is significantly clustered.

**Table 2:** The nearest neighbor index and spatial distribution pattern of the traditional villages in the PRXREB.

\textit{n}	$\overset{―}{\text{\textbackslash{}textit\{D}}{\}}_{\text{\textbackslash{}textit\{O}}\}$(km)	$\overset{―}{\text{\textbackslash{}textit\{D}}{\}}_{\text{\textbackslash{}textit\{E}}\}$(km)	\textit{R}	z-score	p-value	Distribution Pattern
120	12.66	27.14	0.47	−11.18	0.000	clustered

**Fig 3:**
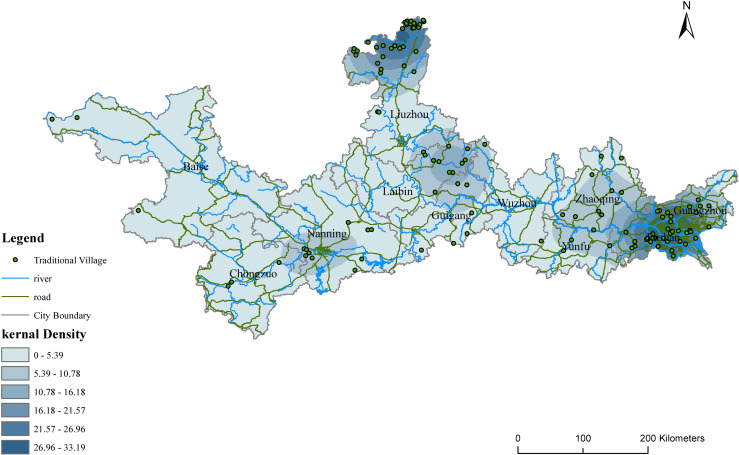
Kernel density distribution of traditional villages in the PRXREB. (Source of base map: the open source map data service provided by the National Platform for Common GeoSpatial Information Services (https://www.tianditu.gov.cn/).

Further, the kernel density estimation was used to visualize the distribution patterns of the traditional villages in the PRXREB (Fig 3). The result shows that the spatial distribution of the traditional villages in the PRXREB is extremely unbalanced. The spatial distribution of the traditional villages in the PRXREB is sparse, with an average density of 6.8 villages per 10,000 km^2^, which is much lower than that of 7.10 villages per 10,000km^2^ nationwide. Furthermore, four main clusters were identified, as shown in Fig 3. The largest cluster of traditional villages is located in Foshan, west of Guangzhou, southeast of Zhaoqing, and northeast of Fuyun, with a core density of 26.96–32.35 villages per 10,000 km^2^. The second cluster is located in the north of Liuzhou. The traditional villages in the east of Laibin, north of Guigang, and northwest of Wuzhou form the third cluster, and the last cluster is located in the southwest of Naning. More importantly, the four clusters are mostly distributed on the fringes of the cities.

## 4. Influencing factors on village distributions in the PRXREB

Natural and socioeconomic factors significantly influence the spatial pattern of the traditional villages in the PRXREB. After reviewing the relevant literature and considering data availability [4,38], five natural and eight socioeconomic factors were selected for analysis. The five natural factors were elevation, slope, aspect, distance to a road (Road), and distance to a river (River). The eight socioeconomic factors were population (Pop), GDP, primary industry (PI), secondary industry (SI), tertiary industry (TI) in GDP, per capita GDP (PGDP), the per capita disposable income of urban residents (PIUR), and the per capita disposable income of rural residents (PIRR). The statistical information of each factor is shown in S2 and S3 Tables.

### 4.1. Natural factors

#### 4.1.1. Elevation.

**Fig 4:**
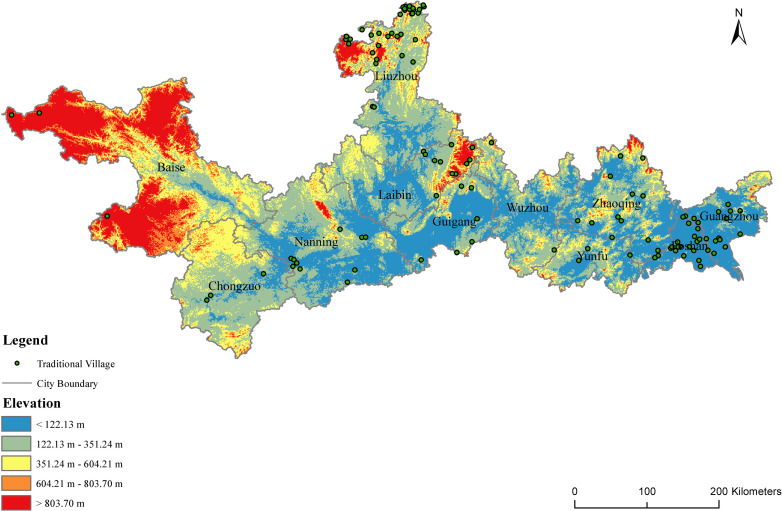
The distribution of the elevation of traditional villages in the PRXREB. (Source of base map: the open source map data service provided by the National Platform for Common GeoSpatial Information Services (https://www.tianditu.gov.cn/).

Elevation is a critical factor in the evolution of the traditional villages in the PRXREB. It affects soil characteristics, water features, and climate, which, in turn, influence the villages’ spatial arrangements and their agricultural and lifestyle practices. Based on DEM, the altitudes of the traditional villages in the PRXREB are displayed in Fig 4. The result indicates that the average elevation of the economic belt reaches 254.08 meters. As the elevation increases, fewer traditional villages exist. There are 45 traditional villages distributed in low mountains and hills, accounting for 37.5% of the traditional villages in the PRXREB. The regional environment where these 45 traditional villages are located has various geographic advantages, such as a good environment and climate, a low topographic relief amplitude, a simple landform, and superior agricultural development conditions, which were conducive to the formation and development of the traditional villages.

**Fig 5:**
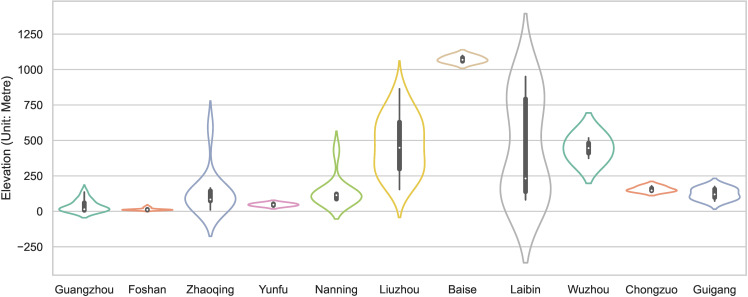
The elevation of traditional villages in the PRXREB.

Generally, the elevation of the traditional villages in the seven cities of the Guangxi Zhuang Autonomous Region is higher than that in the four cities of Guangdong Province (Fig 5), among which Baise has the highest elevation, exceeding 1 km. However, the general central tendency of the village elevation in the seven cities in Guangxi is lower than that in the four cities in Guangdong. In Guangxi, the central tendency of the traditional villages in Nanning and Chongzuo is relatively higher, while the dispersion degree of the elevation of the villages in Laibin and Liuzhou is larger. The distribution of the elevation of the traditional villages located in Guangzhou, Laibin, Guigang, and Nanning is strongly skewed. Specifically, the traditional villages in the three cities Guangzhou, Laibin, and Guigang present a left-skewed distribution, which represents the elevation of most traditional villages that have a median elevation higher than all the traditional villages in those cities. Meanwhile, the traditional villages in Nanning show a right-skewed distribution.

#### 4.1.2. Slopes.

**Fig 6:**
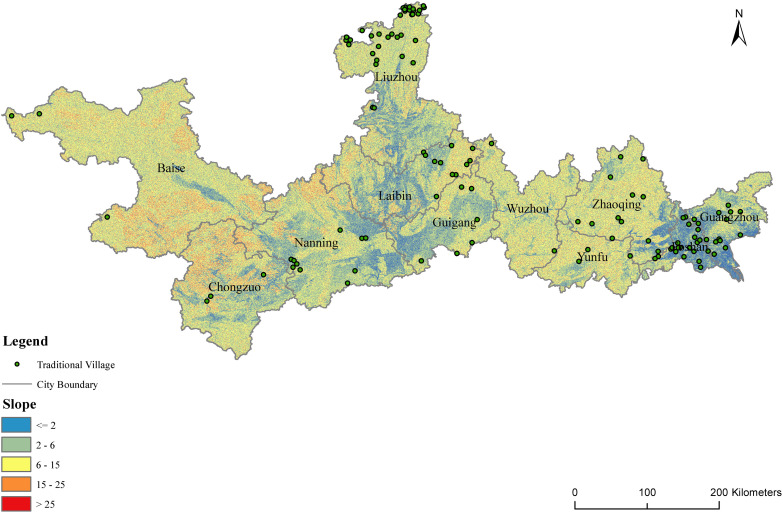
The distribution of the slopes of traditional villages in the PRXREB. (Source of base map: the open source map data service provided by the National Platform for Common GeoSpatial Information Services (https://www.tianditu.gov.cn/).

**Fig 7:**
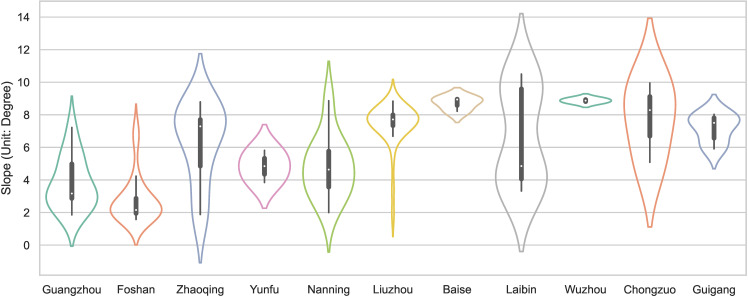
The slopes of traditional villages in the PRXREB.

Slopes affect natural airflow, sunshine duration, and radiation intensity, which impact the site selection, spatial layout, orientation, and ecological environment of traditional villages and residential houses. Based on the digital elevation model (DEM), the slopes of the PRXREB were extracted and divided into five categories [39] which is shown in Table 3. Finally, the distribution of the slopes and the number of traditional villages in the PRXREB were determined, and they are shown in Figs 6 and 7.

**Table 3:** Slope classification.

Range	≤2∘	(2∘ 6∘]	(6∘ 15∘]	(15∘ 25∘]	>25∘
**Topography**	flat slope	gentle slope	slope	steep slope	extremely steep slope

Fig 7 shows that most traditional villages in the PRXREB are located in low-slope areas. Specifically, there are 12 traditional villages distributed in flat areas of the PRXREB, with a slope below 2°, accounting for 10% of the total number of traditional villages. No traditional village is located in areas with a slope of more than 15°. The environment of the areas with a slope greater than 15° is terrible and not suitable for site selection or farming activities, with poor soil, intensive erosion, serious water loss, and a fragile ecosystem. Generally, the relief amplitude of the traditional villages in the four cities of Guangdong is smaller than that of the traditional villages in the seven cities of Guangxi. The average slope of the traditional villages in the four cities of Guangdong is 4°, and the average slope of the traditional villages in the seven cities of Guangxi is 7°. Among the 11 cities in the PRXREB, most of the traditional villages in Guangzhou and Foshan are distributed in areas below 5°, most of the villages in Liuzhou and Wuzhou are distributed in areas between 7° and 9°, and the three traditional villages in Baise are all distributed in areas above 8°. The interquartile range of the traditional villages in Baise, Yunfu, Foshan, and Wuzhou is smaller. However, the interquartile range of Laibin, Zhaoqing, and Liuzhou is larger. Guangzhou, Foshan, Nanning, and Laibin show a left-skewed distribution, and Zhaoqing, Yunfu, Chongzuo, and Guigang show a right-skewed distribution.

#### 4.1.3. Relief degree of land surface (RDLS).

**Fig 8:**
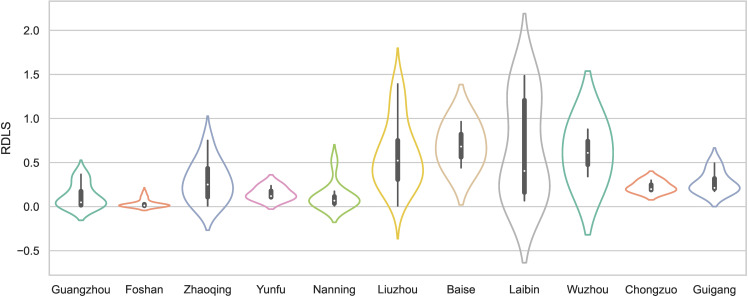
The RDLS of traditional villages in the PRXREB.

RDLS refers to the maximum relative elevation difference within a certain area, which can reflect the environmental conditions. Based on [40], we calculated the RDLS of the traditional villages as follows:


$$\begin{array}{c} RDLS=\left\{\left[Max(H)-Min(H)\right]\times \left[1-P(A)\text{/}A\right]\right\}\text{/}500 \end{array}$$ (7)

where AVG(H) is the average elevation of the traditional village, Max(H) and Min(H) represent the highest and lowest elevations of the traditional village, P(A) is the area of flat land in the traditional village, and A is the total area of the traditional village. To characterize the regional differences in RDLS in China, we chose a height of 500 m in low hilly areas as the standard height for evaluating RDLS. The value of RDLS indicates the multiple of the surface relief to the height of the base mountain. If the value is less than 1, then the relief land surface is lower than the height of the base mountain. Fig 8 shows that most traditional villages in the PRXREB are located in low-RDLS areas. Specifically, 55% of traditional villages are located in areas with an RDLS less than 0.25. Areas with a low RDLS are conducive to building houses and making it easier for people to travel and live. Only 10 traditional villages have an RDLS greater than 1, of which 4 villages are located in Laibin and the remaining 6 villages are located in Liuzhou.

#### 4.1.4. Road and transportation.

**Fig 9:**
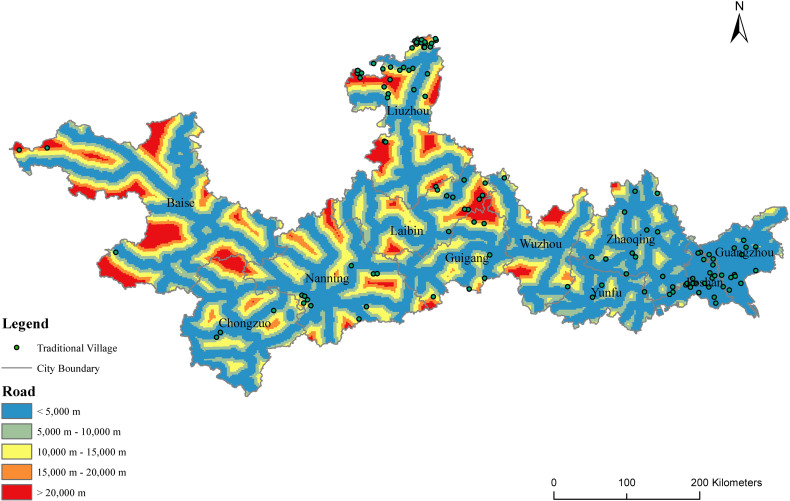
The distribution of distance to major roads in the PRXREB. (Source of base map: the open source map data service provided by the National Platform for Common GeoSpatial Information Services (https://www.tianditu.gov.cn/).

Road network density is closely related to the level of regional economic development. Roads and transportation provide basic support for identifying the pattern of regional economic development. The distribution of major roads and rivers in the PRXREB is shown in Fig 9. It can be seen in Fig 9 that there are obvious differences in the distribution of the traditional villages in Guangxi and Guangdong. The traditional villages in Guangxi are mainly distributed in areas with a low road density, while the traditional villages in Guangdong are located in areas with a high road density. A higher road density near a village means that it has high transportation convenience and is easier to communicate with the outside world.

**Fig 10:**
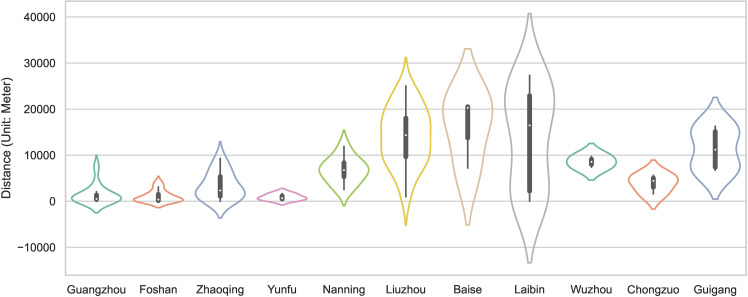
The distance between traditional villages and major roads in the PRXREB.

To quantify the transportation convenience of the traditional villages in the PRXREB, the shortest distance from each village to major roads was calculated (Fig 10). It should be noted that, since the railways and expressways in China can only be accessed from a small number of stations, the major roads in our study only include national highways, provincial highways, and urban secondary roads and above. The results show that the average shortest straight-line distance between the traditional villages and major roads is 7.31km in the PRXREB. The average shortest distance from the traditional villages to the main road in the four cities of Guangdong is within 5km, which is generally less than the distance between the seven cities of Guangxi, indicating that the transportation convenience of the traditional villages in Guangdong is better than that of the traditional villages in Guangxi. Inconvenient transportation has prevented the traditional villages in Guangxi from being influenced by the culture of the outside world, and it has protected the local historical buildings, traditional customs, and cultural heritage. However, convenient transportation has enabled the integration of multiculturalism in Guangdong villages, promoted the development of the local economy, and improved the living environment and the quality of life of local villagers. In addition, the interquartile range of the average shortest distance from the traditional villages to the major roads in Guangzhou, Foshan, Yunfu, Wuzhou, and Chongzuo is small. The largest interquartile range is in Laibin. Guangzhou, Foshan, Zhaoqing, Yunfu, Baise, Laibin, Chongzuo, and Guigang have a strongly skewed distribution in terms of the distance from the traditional villages to major roads. Among the eight cities, Guangzhou, Foshan, Zhaoqing, and Yunfu in Guangdong show a left-skewed distribution, and Baise, Laibin, Chongzuo, and Guigang in Guangxi show a right-skewed distribution.

#### 4.1.5. Rivers.

**Fig 11:**
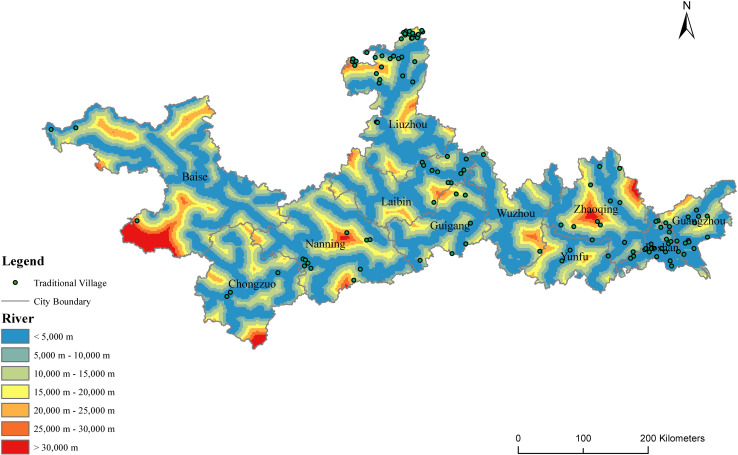
The distribution of distance to major rivers in the PRXREB. (Source of base map: the open source map data service provided by the National Platform for Common GeoSpatial Information Services (https://www.tianditu.gov.cn/).

Rivers provide basic material support for human production and life, and they are also an important factor that may affect the layout of the location, landscape, development scale, and spatial distribution of the traditional villages in the region. According to Fig 11, the traditional villages are mostly distributed along the main streams and tributaries of rivers, which is more notable in the Pearl River Basin and more clustered in the Pearl River Delta.

**Fig 12:**
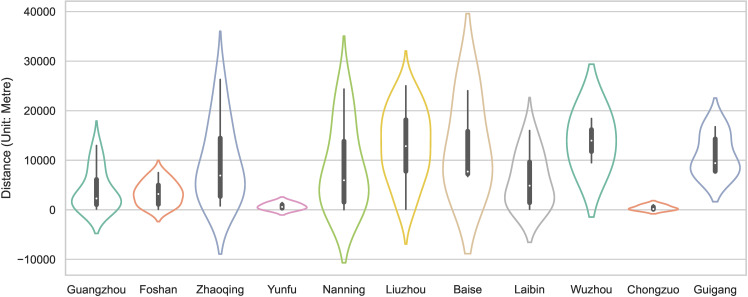
The distance between traditional villages and major rivers in the PRXREB.

According to the shortest straight-line distance analysis of the major rivers in the PRXREB (Fig 12), there are 50, 25, 13, 15, 7, and 2 traditional villages distributed within 5km, 10km, 15km, 20km, 25km, and 30km from the main river, accounting for 44.64%, 22.32%, 11.61%, 13.39%, 6.25%, and 1.79%, respectively. In Yunfu and Nanning, the interquartile range of the distance between village roads and major rivers is small. However, in Zhaoqing, Nanning, Liuzhou, Baise, and Laibin, the interquartile range of the distance between village roads and major rivers is large. Guangzhou, Zhaoqing, Yunfu, Nanning, Baise, Chongzuo, and Guigang have a strongly skewed distribution in terms of the distance from traditional villages to major rivers, and they present a left-skewed distribution. More than half of the villages are less than 10 kilometers away from the main river in a straight line; this shows that ancient villagers preferred to build their residences near rivers, which could solve not only the domestic water problem but also the inconvenient road transportation problem.

Rivers in Chinese history determined the growth of population centers. Rivers provided the necessary water source for ancient Chinese agricultural production and supported the production and life of a large population; rivers were important channels for transportation, commodity circulation and cultural exchange, promoting economic prosperity and population mobility; riverside areas were often flat and open, and were mostly the economic, cultural and political centers of ancient China, making them more likely to attract population concentration.

### 4.2. Socioeconomic factors

#### 4.2.1. Population distribution.

**Fig 13:**
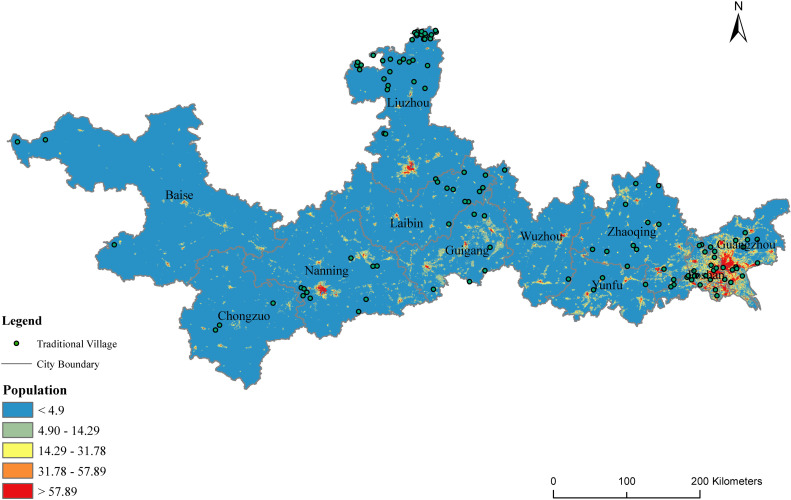
Population density distribution of the PRXREB in 2020. (Source of base map: the open source map data service provided by the National Platform for Common GeoSpatial Information Services (https://www.tianditu.gov.cn/).

The population is an important cornerstone for the long-term existence and sustainable development of traditional villages. A population density distribution map of the PRXREB in 2023 is shown in Fig 13. The result shows that most of the population was concentrated in urban areas and that the population density in Guangzhou, Foshan, and Nanning was relatively high. According to the demographic statistics released by the government (S2 Table), the populations of Guangzhou, Foshan, and Nanning were 18.83 million, 9.62 million, and 8.94 million in 2023, accounting for 29.97%, 15.30%, and 14.23% of the total population in the PRXREB, respectively.

**Fig 14:**
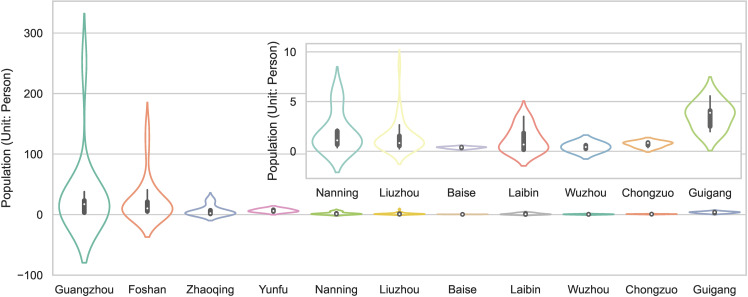
The population densities of traditional villages in different cities.

Previous studies used the total population of cities to analyze traditional villages [38]. However, there were large differences in the population data of various traditional villages, as well as between the rural population and the entire city population. Therefore, the population density of a city cannot accurately represent the population of traditional villages. To obtain the population data of each traditional village, first, the point of the traditional village was taken as the center; then, a circular area with a radius of 2 km was obtained; and, finally, the average population density in this circular area was calculated as the population density of the traditional village. The minimum distance between villages in the PRXREB is about 2 km. If the radius exceeds 2 km, it could lead to overlapping coverage areas between some villages, so we choose 2 km as the radius of the circular area. Finally, the population densities of the traditional villages in the different cities are shown in Fig 14. It can be concluded from Fig 14 that the population density of the traditional villages in the cities of Guangdong is higher than that of the traditional villages in the cities of Guangxi. The population density and interquartile range of Guangzhou are the highest, followed by those of Foshan and Yunfu. The population density of the traditional villages in Baise, Wuzhou, and Chongzuo is less than 2 people per 10,000 m^2^.

The population was the basis for the formation of settlements. A sufficient population provided conditions for the formation and inheritance of villages [41]. However, with the development of the economy, the population began to migrate to urban areas, so the population density in urban areas became relatively high. This also indicates that the current traditional villages far away from cities faced the problems of population loss and aging, which brought challenges to the inheritance of traditional villages.

#### 4.2.2. Social economy.

Socioeconomic factors significantly influence the preservation and evolution of the traditional villages in the PRXREB. It is evident that there is a relationship between the economic development of the cities in the economic belt and the spatial distribution of the traditional villages. Table 4 illuminates those economically stronger cities such as Guangzhou, Foshan, Nanning, and Liuzhou, which lead in terms of economic metric, there is a higher concentration of traditional villages, representing 67.5% of the total. Conversely, regions with slower urbanization and less intensive development, such as Zhaoqing in Guangdong and Laibin in Guangxi, demonstrate a more robust preservation of traditional villages. Despite their lower GDP contribution (9.84% of the total of the 11 cities), these areas account for 19.17% of the traditional villages, emphasizing a correlation between the pace of economic development and village preservation.

**Table 4:** Statistics on national economy and social development of cities in the PRXREB in 2023.

City	GDP (CNY billion)	PI (CNY billion)	SI (CNY billion)	TI (CNY billion)	PGDP (CNY)	PIUR (CNY)	PIRR (CNY)
Guangzhou	30355.73	317.78	7775.71	22262.24	161634	80501	38607
Foshan	13276.14	228.78	7513.72	5533.64	138215	68643	41423
Zhaoqing	2792.51	498.94	1154.56	1139.01	67614	39679	24809
Yunfu	1207.42	222.55	387.29	597.58	50382	33628	21909
Nanning	5469.06	636.4	1194.58	3638.08	61338	44469	20369
Liuzhou	3115.86	298.68	1232.62	1232.62	74679	44177	19811
Wuzhou	1490.77	221.2	601.3	668.27	52640	40181	18680
Guigang	1573.49	287.91	504.83	780.75	36321	39145	20829
Baise	1849.81	331.01	849.4	669.41	52321	39154	16972
Laibin	981.41	208.57	334.45	438.39	47538	41702	17521
Chongzuo	1117.56	214.92	421.15	481.49	53939	39502	17918
PRXREB	63229.76	3466.74	21969.61	37441.48	796621	510781	258848

#### 4.2.3. Regional culture.

Regional culture and customs had a profound impact on the formation and development of traditional villages. Lingnan culture is the common cultural foundation of Guangdong and Guangxi, creating the same history, connection, language, and customs in the PRXREB, and this promoted the complementarity and integration of regional economies and improved the competitiveness of the PRXREB. Lingnan culture was divided into Guangfu culture, Chaoshan culture, and Hakka culture, and its spirit “to be a pioneer” promoted the formation and development of traditional villages with profound cultures and distinct regional features. Moreover, the PRXREB was inhabited by twelve ethnic groups, among which the Hans, the Miaos, the Yaos, the Dongs, etc., were the aborigines in the region. The population of minorities accounts for more than 80% of the residents in Guangxi. The integration and exchange of the cultures of the Han nationality and the ethnic minorities formed the unique cultures of the national-level traditional villages.

Historically, the site selection process of traditional villages in the PRXREB has been shaped by factors such as resource access, trade routes, and cultural heritage. Villages were typically located along riverbanks or near water sources to ensure access to essential resources for agricultural production and daily life. The site selection was closely influenced by natural resource factors, including the abundance of water sources, soil fertility, transportation convenience, and climate conditions. Additionally, as transportation hubs connecting different regions, the establishment of these villages was closely linked to the formation of important trade routes. These villages not only facilitated the exchange of goods and culture but also stimulated local economic development. Over time, they developed unique socio-economic structures.

Furthermore, as carriers of cultural transmission, the site selection and development of villages were also influenced by cultural factors such as religious beliefs, family settlements, and the transmission of customs. These cultural elements not only shaped the spatial layout of the villages but also influenced the social relationships and economic activities of their inhabitants.

With the progress of modernization, the socio-economic conditions in the PRXREB have undergone significant changes. Many traditional villages are now facing challenges such as population decline, resource depletion, and economic stagnation. However, some villages have maintained their vitality by adapting to new economic opportunities, such as emerging industries in tourism, specialized agriculture, and handicrafts, thereby successfully achieving economic transformation. In addition, the improvement of modern infrastructure (such as transportation, communication, and public service facilities) has had a profound impact on the survival and development of traditional villages. Villages that effectively leverage new infrastructure are better positioned to integrate into the modern economic system, while those that fail to adapt—due to factors such as remote locations, poor transportation access, or population loss—are at risk of further decline.

### 4.3. Factor detection of spatial pattern

The factors should be classified before using geographic detectors [37]. Arbitrary classifications may fail to characterize the relationship between factors and the spatial patterns of traditional villages. After reviewing the literature [38,42], the natural break method was chosen to classify all economic factors, Ele, Slope, and RDLS, and the manual method was chosen to classify the remaining factors. The classification results of all factors are shown in Table 5.

**Table 5:** The classification results of all influencing factors.

Category	Factor	Classes	Range	Cutting values
**Natural factors**	**Ele**	5	[4.25, 1099]	72.50, 231.64, 475.29, 731.15
	**Slope**	3	[1.57, 10.50]	2, 6
	**RDLS**	5	[0.01, 1.49]	0.13, 0.32, 0.53,0.99
	**Road**	5	[0.96, 27351.3]	5000, 10000, 15000, 20000
	**River**	6	[24.75, 26293.8]	5000, 10000, 15000, 20000, 25000
**Socioeconomic factors**	**Pop**	5	[0.08, 251.90]	4.92, 17.20, 51.86, 145.88
	**GDP**	6	[981.41, 30355.73]	981.41, 1207.42, 1849.81, 5469.06, 13276.14
	**PI**	5	[208.57, 636.4]	214.92, 228.78, 298.68, 331.01
	**SI**	5	[334.45, 7775.71]	421.15, 601.30, 849.40, 1232.62
	**TI**	5	[438.39, 22262.24]	481.49, 780.75, 1232.62, 5533.64
	**PGDP**	6	[36321, 161634]	36321, 47538, 53939, 67614, 74679
	**PIUR**	5	[33628, 80501]	33628, 40181, 41702, 44469
	**PIRR**	5	[16972, 41423]	18680, 20369, 21909, 24809

#### 4.3.1. The factor detector.

The importance of the influencing factors on the spatial pattern of traditional villages is shown in Table 6. The q value represents the importance of the influencing factor, and the P value determines the result of the hypothesis test. The smaller the P value, the more significant the result.

**Table 6:** The factor detection result of geographic detector.

	Natural factors					Socioeconomic factors							
**Ele**	**Slope**	**RDLS**	**Road**	**River**	**Pop**	**GDP**	**PI**	**SI**	**TI**	**PGDP**	**PIUR**	**PIRR**
*q*	0.149	0.032	0.095	0.179	0.010	0.105	0.348	0.253	0.188	0.168	0.438	0.499	0.262
\textit{P}	0.007	0.695	0.067	0.000	0.999	0.978	0.003	0.000	0.381	0.043	0.000	0.000	0.004

Table 6 shows that most factors had significant influences on the spatial pattern of the traditional villages in the PRXREB (P < 0.05). Most socioeconomic factors had significant influences on the spatial pattern of the traditional villages in the PRXREB. The PIUR factor had the largest influence, explaining 0.499 of the spatial patterns of the traditional villages. The following socioeconomic factors were PGDP, GDP, PIRR, PI and TI, whose contributions were 0.438, 0.348, 0.262, 0.253, and 0.168, respectively. The natural factors of distance to a road and elevation explained only 0.179 and 0.149, respectively.

In addition, natural factors were also related to the spatial pattern of the traditional villages in the PRXREB, but their influence was smaller than that of the socioeconomic factors. The factor detector illustrated that the distance to a road (0.179) was the most influential natural factor, followed by elevation (0.149). Generally, road density was positively correlated with the economy. Economically developed regions built more roads to improve transportation convenience, so the distance to the main road was smaller, and vice versa.

Specifically, urban residents’ income emerged as the most influential factor shaping the distribution of the traditional villages. Increased incomes in urban areas boost consumption in sectors such as services and tourism. The traditional villages in the PRXREB, renowned for their rich ethnic minority cultures, unique regional traits, and abundant tourism resources, attract urban visitors. This trend has spurred the growth of tourism-focused products in rural areas, enhancing both agricultural and cultural tourism, which, in turn, has boosted the tertiary and primary sectors, raising rural incomes. Consequently, factors such as GDP, PI, TI, PGDP, PIUR and PIRR are pivotal in shaping the distribution of these villages. Moreover, the growth of tourism in these villages necessitates high-quality ecological environments and infrastructure, particularly in transportation, making road accessibility a key factor.

#### 4.3.2. The interactions of influencing factors.

**Fig 15:**
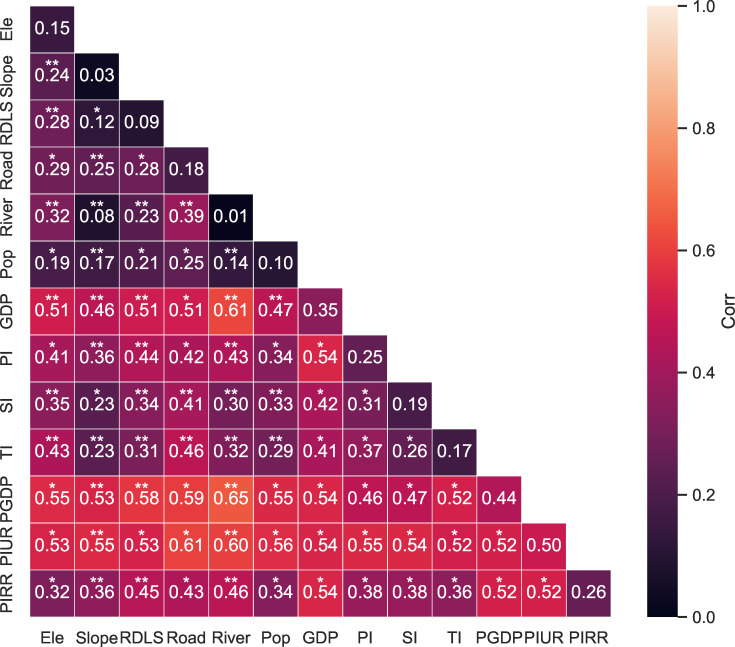
The interaction detection results of geographic detector. (“*” indicates bi-enhancements, and “**” indicates nonlinear enhancements).

Further analysis revealed that the interplay between various factors either amplified their collective impact on the spatial distribution of the traditional villages (Fig 15). It was found that interactions between natural and socioeconomic factors significantly strengthened their collective influence, with socioeconomic interactions proving to be more potent than natural ones. Notably, PIUR, when combined with other factors, displayed interaction values surpassing its individual impact, with all exceeding 50% in explanatory power. This suggests that PIUR’s interaction with other factors doubly enhances its impact on the spatial distribution of the traditional villages. The combined effect of PGDP and distance to a river was particularly significant (0.65), indicating a major influence on the spatial distribution of these villages. Interactions involving PGDP, PIUR, River, Road, GDP, and PI were notably strong. Even factors that individually had a lesser impact were significantly elevated through these interactions. Even the single factors with a low impact in the factor detector had a significantly improved influence after interacting with the above four factors. These results indicate that the traditional villages rely on their advantages to develop tourism, promote the development of the tertiary industry, increase the income of villagers, and drive the growth of GDP. After the local government became rich, it invested more funds in infrastructure construction, especially in road construction, thereby improving transportation convenience and the living environment, with the aim of attracting more tourists. This created greater profits and better promoted local economic development. Therefore, the effects of those factors were enhanced.

These findings indicate that the spatial pattern of the traditional villages in the PRXREB is not merely a consequence of individual factors operating in isolation but rather is shaped by their combined interactions. Thus, when devising development strategies for these villages, it is crucial to consider the synergistic effects of multiple factors.

The PRXREB is a significant economic region in southern China, and the distribution and development of traditional villages here are deeply influenced by both economic and environmental factors. Economic factors are the main drivers of traditional village distribution. The level of economic development directly impacts resource allocation and population movement. Traditional villages in economically developed regions tend to receive more investment and infrastructure development, attracting immigrants and diversifying economic activities. Furthermore, with ongoing economic transformation, improvements in transportation, communication, and utilities are facilitating changes in the industrial structure and economic models of traditional villages. This transformation not only alters their economic foundation but also reshapes their spatial layout.

Natural factors also play a crucial role in the spatial distribution of traditional villages. Areas rich in water resources and fertile soil have fostered unique agricultural practices, making them prime locations for the establishment and development of traditional villages in the PRXREB. With increasing environmental awareness, the protection of ecological environments and the development of green economic strategies provide a foundation for the long-term sustainability of these villages. Additionally, the region’s favorable warm and humid climate supports diverse agricultural practices, further attracting farmers and influencing the spatial distribution of villages.

Influenced by both economic and natural factors, traditional villages in the PRXREB exhibit unique spatial patterns and development models. These experiences offer valuable lessons for encouraging other traditional villages to diversify their economic activities, invest in infrastructure, protect the ecological environment, and achieve sustainable development.

## 5. Discussion

Traditional villages are vital in the cultural aspect of China’s rural revitalization efforts [32]. The study of spatial distribution patterns and their underlying principles forms a cornerstone of geographical research [43]. This research has revealed diverse spatial distributions among traditional villages in the PRXREB, emphasizing the impact of economic, nature and transportation factors. These findings corroborate existing research that notes the ‘uneven and insufficient’ development within these regions.

The formation of traditional villages is a long and complex historical process, which often takes hundreds of years to accumulate and evolve. During these hundreds of years, natural environmental factors such as topography, climate conditions, and natural resources will affect the spatial layout, architectural style, and production and living styles of traditional villages. Socioeconomic factors such as population growth, economic development, and traffic changes will also promote the temporal and spatial evolution and transformation of traditional villages. Cultural traditional factors such as religious beliefs, folk customs, and family systems constitute the spiritual core of traditional villages, inheriting the memory of history and the essence of culture. The interaction of various factors such as natural environment, social economy, and cultural traditions has jointly shaped the unique style and profound heritage of traditional villages.

The analysis reveals that harsh natural conditions indirectly influence village distribution by constraining regional economic development, which is consistent with the results of previous studies [44]. Specifically, challenging environmental features can directly impede agricultural productivity and other economic activities. These natural limitations typically result in poorer local economies, which affect population density and residential choices, thereby altering the spatial distribution of traditional villages. Although natural conditions are primary constraints, the actual influences on village distribution stem from the resulting economic and social conditions, such as limited employment opportunities and inadequate infrastructure [45].

Despite the potential of modern technology to mitigate some adverse effects of natural conditions, these improvements often necessitate significant economic investment and technical support [12]. In economically underdeveloped regions, the capacity to adapt to and modify the environment is limited, and the adverse impacts of natural conditions remain significant. Consequently, a complex interaction between natural and socio-economic factors not only affects the immediate spatial distribution of villages but also shapes the region’s social structure, cultural landscape, and sustainable development through its influence on long-term economic paths [46].

In conclusion, the interplay between natural environments and socio-economic factors forms a complex system essential for studying the spatial distribution of traditional villages. Future research should employ multivariate statistical methods to delve deeper into the correlations and mechanisms of these factors, enhancing our understanding and aiding in the preservation of these cultural and historical heritages [47].

Developing rural tourism uniquely tailored to these areas is seen as an eco-friendly strategy for conserving traditional rural landscapes [10], especially in less developed regions of China. This strategy plays a pivotal role in shaping human settlement environments, reflecting dynamic changes in economic growth and material culture brought about by rural tourism. However, the rapid expansion of distinct rural tourism, often undertaken without sufficient planning, is encroaching upon ecological, productive, and living spaces [48], leading to uneven distribution of public resources, ecological degradation, and rampant commercialization. These issues amplify the conflicts between uniformity and uniqueness in rural settings, potentially destabilizing traditional rural environments and endangering the preservation of rural cultural heritage [47,49]. It is crucial to balance tourism development with the preservation of traditional rural characteristics to mitigate new conservation challenges related to traditional architecture and culture.

## 6. Conclusions

Based on a spatial analysis and geographical statistics, the spatial patterns of traditional villages in the PRXREB are quantitatively analyzed and determined, and the influences of natural and socioeconomic factors alone and in combination on traditional villages are explored. Finally, the measures of traditional village protection and revitalization are discussed. The main conclusions are as follows:

(1) Traditional villages in the PRXREB exhibit a spatial pattern characterized by four cluster centers. However, their distribution is notably unbalanced and clustered, particularly concentrated in Foshan, Guangzhou within Guangdong, and Liuzhou within Guangxi.
(2) Traditional villages in seven cities of Guangxi generally have higher elevations compared to those in four cities of Guangdong. Most villages are located in low mountain and hilly regions with slopes less than 10° and situated near major rivers and roads, with nearly 80% of Guangdong’s villages within 5km of major rivers and 95% near major roads.
(3) Natural conditions indirectly affect village distribution by affecting regional economic development. Thus, the results of geographical detectors indicate that the influence of socioeconomic factors is stronger than that of natural factors. PIUR is the most influential factor. Interactions between natural and socioeconomic factors enhance each other’s influence on the spatial patterns of the traditional villages in the PRXREB. It is evident that the interplay of various factors, rather than their independent actions, shapes the spatial distribution of these villages in the PRXREB.
(4) Some traditional villages in Guangxi, characterized by low population density and inadequate transportation, are at risk of disappearing. It is recommended that government initiatives focus on leveraging the unique cultural and ecological attributes of these areas to promote rural tourism, increase employment opportunities, and protect the tangible and intangible heritage of these villages.

However, the study has limitations that future research should address. Future research should leverage advanced methods such as big data technology to gather more detailed, micro-level data. This could involve analyzing mobile signal data, social media data, and POIs to gain insights into tourism trends, population movements, and settlement densities, thus enriching data sources and enhancing the study’s explanatory depth [50]. Additionally, there is a gap in knowledge regarding the interplay of various factors at specific rural sites [44]. Shifting the focus from urban to rural areas, especially under rural revitalization strategies, is essential to examine the spatial interconnections between rural tourism and living environments. Furthermore, expanding the temporal dimension in the study of influencing factors will help to uncover the spatiotemporal patterns of traditional rural areas and the intensity of various spatiotemporal influences.

## Supporting information

S1 Table Chinese national traditional villages in PRXREB. (XLSX)

S2 Table Statistics of national economic and social development of cities in PRXREB in 2023. (XLSX)

S3 Table Statistics of the influencing factors. (XLSX)
